# Ancestry informative genetic variants associated with tobacco metabolic and detoxification capacity measured by 4-(methylnitrosamino)-1-(3-pyridyl)-1-butanol (NNAL) among smokers

**DOI:** 10.1007/s00204-026-04505-w

**Published:** 2026-07-14

**Authors:** Oladimeji Lanade, Zhongxuan He, Yan Zhou, Elizabeth Blackman, Denise Gibbs, Sharon Harrison, Karthik Devarajan, Mark Andrake, Roland L. Dunbrack, Gerald Nkogbu, Aditi Wagh, Michael Slifker, Cherie Erkmen, Freda Patterson, Aditi Satti, Heather-Dawn Lawson-Myers, Robin Roberts, Don Mitchell, Jeffery C. Liu, Joel Erblich, Camille Ragin

**Affiliations:** 1 Cancer Prevention and Control Program, Fox Chase Cancer Center-Temple Health, Philadelphia, PA USA; 2 Department of Biostatistics & Bioinformatics, Fox Chase Cancer Center-Temple Health, Philadelphia, PA USA; 3 Cancer Signaling and Microenvironment Program, Fox Chase Cancer Center-Temple Health, Philadelphia, PA USA; 4 Non-Therapeutic Trial Unit, Fox Chase Cancer Center – Temple Health, Philadelphia, PA USA; 5 Lewis Katz School of Medicine, Temple University, Philadelphia, PA USA; 6 College of Health Sciences, University of Delaware, Newark, DE USA; 7 Liguanea Family Dental, Kingston, Jamaica; 8 School for Clinical & Medical Research, University of the West Indies, Nassau, Bahamas; 9 Us TOO - Grand Bahama Prostate Cancer Support Group, Freeport, Bahamas; 10 Hunter College, City University of New York, New York, NY USA; 11 African Caribbean Cancer Consortium, Philadelphia, PA USA

**Keywords:** NNAL, Tobacco metabolism, Ancestry informative markers

## Abstract

Tobacco use remains the leading preventable cause of cancer mortality worldwide, with marked disparities in cancer incidence and outcomes across racial and ethnic groups. This study investigates the genetic determinants of tobacco carcinogen metabolism among smokers, focusing on ancestry-informative markers (AIMs) near genes involved in metabolic activation (Phase I), detoxification (Phase II), and transport (Phase III) of tobacco-specific carcinogens. Using data from 274 smokers recruited in the United States and the Caribbean, we measured urinary NNAL (a metabolite of NNK) to classify metabolic phenotypes and performed genotyping with a customized array enriched for AIMs. Most participants (94%) were classified as poor metabolizers of NNAL, with a higher prevalence among Black/African American smokers. Multivariable analyses, after adjusting for age, sex, assay batch, and number of cigarettes smoked per day, identified 14 AIMs near Phase I, II, and III pathway genes significantly associated with NNAL metabolizer phenotype, with African ancestry alleles conferring increased odds of poor metabolism. Functional annotation revealed that most significant AIMs overlapped regulatory regions, and the *CYP3A5* rs776746 variant demonstrated structural disruption likely to impair enzymatic activity. Majority of participants had high African ancestry and were poor NNAL metabolizers, consistent with the observed association between African ancestry and poor NNAL metabolism. These findings highlight the biological basis for tobacco-related cancer disparities and underscore the importance of integrating genetic ancestry and metabolic phenotyping in risk assessment and personalized prevention strategies for tobacco-associated cancers.

**Supplementary Information:**

The online version contains supplementary material available at https://doi.org/10.1007/s00204-026-04505-w.

## Introduction

Worldwide, tobacco use accounts for the largest avoidable cause of cancer mortality and is reported to be responsible for 26% of total cancer deaths in 2019 (36% in men and 12% in women). Similarly, in the United States (U.S.) it is estimated that cigarette smoking causes about 30% of all cancer deaths (Islami et al. 2024). The association between tobacco use and its role in the etiology of certain cancers is well established (Boyle 1997; Cresanta 1992; Hammond and Horn 1954; Park et al. 2014; Poirier et al. 2016; Sasco et al. 2004; Watson and Conte 1954, 1955; Wynder 1954). The use of tobacco has been linked to increased risk of at least 17 cancers, including acute myeloid leukemia, cancers of the oral cavity and pharynx, larynx, lung, esophagus, pancreas, uterine cervix, kidney, bladder, stomach, colorectum, and liver (Islami et al. 2024). Our research primarily focused on lung cancer and head and neck cancers, two cancer groups that account for a significant share of tobacco-related cancer cases and deaths.

Although lung cancer’s global impact is evident, its effect on different demographics is not uniform, with notable differences in incidence and mortality rates amongst differing demographics such as race/ethnicity and sex. One striking example is seen between non-Hispanic black and white males in lung cancer. The National Cancer Institute (NCI) reports a 2021 ageadjusted incidence rate per 100,000 of 64.2 among non-Hispanic Black males while reporting a rate of 56.6 among non-Hispanic White males and females combined in the U.S. Similarly, U.S. age-adjusted mortality rates per 100,000 in 2022 were 42.7 among non-Hispanic Black males and 37.5 among non-Hispanic White males. Compared with White Americans, Black Americans also have lower rates of localized disease at diagnosis (18% vs. 22.2%), higher rate of distant disease at diagnosis (55.5% vs. 49.8%), and poorer 5-year overall survival rate (21.7% vs. 19.6%) (National Cancer 2024). This difference in incidence and mortality has been consistently seen for at least two decades between both male and female U.S. populations.

For head and neck cancer, the NCI reported in 2021 an age-adjusted incidence rate per 100,000 of 13.9 among non-Hispanic White persons of both sexes (4.5 in males only, 1.6 in females only) while reporting an age-adjusted incidence rate per 100,000 of 8.5 among non-Hispanic Blacks of both sexes (13.1 in males only, 4.8 in females only). Age-adjusted mortality rates per 100,000 reported in 2022 were 3.0 in non-Hispanic White persons of both sexes (4.5 in males only, 1.6 in females only) and 2.6 in non-Hispanic Black persons of both sexes (4.4 in males only, 1.2 females only) (National Cancer 2024).

Research by our team and others (Erblich et al. 2012; Ramakodi et al. 2017; Schwartz et al. 2009) have raised the intriguing possibility that differences in tobacco-related cancer incidence between Black Americans and White Americans may be attributable to genetic ancestry factors that contribute to poorer metabolism of tobacco-related carcinogens (Berg et al. 2010; Muscat et al. 2005; Richie et al. 1997). Our research focuses specifically on two major groups of potent tobacco carcinogens, N’-nitrosonornicotine (NNN) and 4-(methylnitrosamino)-1-(3-pyridyl)-1-butanone (NNK). These carcinogens are reduced to 4-(Methylnitrosamino)-1-(3-pyridyl)- 1-butanol (NNAL), a potent carcinogen in rats (Belinsky et al. 1990; Rivenson et al. 1988) and have been correlated to lung cancer risk (Ashley et al. 2010; Church et al. 2009; Sarkar et al. 2012; Yuan et al. 2009, 2014). Genetic polymorphisms have been described for a number of Phase I, II/III enzymes involved in the metabolism of tobacco and efflux of carcinogens. Many of these polymorphisms have been linked to phenotypic differences in protein activity or expression and are likely to be a major source of inter-individual variation in susceptibility to tobacco carcinogenesis and thus increased lung cancer risk (Ford et al. 2000; Garte et al. 2007; Kawajiri et al. 1990; Kiyohara et al. 2002; Megaraj et al. 2011; Taioli et al. 1998). Levels of urinary NNAL metabolites can be influenced by polymorphisms in the Phase I and II genes, (Chen et al. 2008; Chung et al. 2013) highlighting the importance of genetic variants in tobacco metabolism and carcinogenesis. Previous studies have reported higher metabolic efficiency of NNAL among White Americans compared to Black Americas (Muscat et al. 2005; Richie et al. 1997), suggesting racial differences in metabolic activation/detoxification of tobacco smoke carcinogens and possible implication of genetic ancestry. In this study we seek to further examine the efficiency of tobacco smoke metabolism among smokers by investigating the association of tobacco metabolism with genetic ancestry. We hypothesize that African genetic ancestry and ancestry-informative variants in tobacco metabolism pathway genes are associated with measures of NNAL metabolism in smokers.

## Methods

### Study population

This study was approved by the Fox Chase Cancer Center Institutional Review Board (IRB). The Cancer Prevention Project of Philadelphia (CAP3) was established by the African Caribbean Cancer Consortium (AC3) and included persons enrolled in the US and in the Caribbean (The Bahamas and Jamaica), aged 18 or older with no known diagnosis of cancer at the time of participation (Blackman et al. 2018). A description of the CAP3 study and its recruitment strategies have been previously described (Blackman et al. 2018). Briefly, both community-based and clinic-based strategies were employed to recruit participants. Community outreach involved engaging with individuals at health fairs, community centers, and senior centers, facilitated by an established network of partnerships with key community- and faith-based organizations in the US and Jamaica. In clinical settings, recruitment was conducted in primary care and specialty clinics within the Temple University Health System (TUHS) as well as a cancer screening clinic in Freeport Bahamas. Participants were asked to complete an interviewer-administered questionnaire. After completing the survey, participants provided biological specimens, including buccal swabs or mouthwash samples and urine, for subsequent genetic and biochemical analyses. For this study only smokers, defined as individuals who were current cigarette smokers or users of other forms of tobacco (e.g., cigars) at the time of enrollment were included (*N* = 274). Current cigarette smokers were defined as persons who reported smoking daily or occasionally. There was one person who reported smoking cigars and was also included.

### Questionnaire data

The questionnaire utilized for this study included the CaPTC-AC3 Epidemiology and Behavioral survey instrument that was culturally tailored for Black populations in the US, Caribbean and Africa (Odedina et al. 2019). Questions were adapted from the 2011 Behavioral Risk Factors Surveillance System Questionnaire and the National Health and Nutrition Examination Survey (BRFSS 2011; NHANES 1999) and details of the development of these culturally tailored measures have been previously described (Blackman et al. 2018; Odedina et al. 2019).

While the questionnaire was designed to gather a wide range of variables on participants’ demographics, health behaviors, family and medical history, this analysis focuses specifically on select demographic variables, such as gender, age, race, educational attainment, marital status, and total household income. Tobacco use behaviors were assessed, including participants’ smoking status (e.g., ever smoker or current smoker), types of tobacco used, and the quantity of tobacco consumed.

### Biospecimen collection and processing

After the administration of the study questionnaire biospecimen collection included mouthwash/buccal samples for DNA extractions and germline genotyping as well as urine for biomarker analyses of tobacco metabolites.

*Mouthwash/buccal samples*: Participants were required to refrain from eating and drinking at least 30 min prior to performing the mouthwash rinses. Each participant was provided a 50 mL Falcon™ tube and a 30 mL specimen cup containing 10 mL of Scope and instructed to gargle while tilting their heads back for 30–60s before spitting it back into the 50 mL tube. Mouthwash samples were transported at room temperature to the laboratory for processing. Samples were centrifuged, and the mouthwash supernatant was removed, and DNA extraction was performed from the oral exfoliated cell pellet using the QIAGEN PureGene DNA extraction protocol (Qiagen 2014). DNA quality was assessed for each sample and tested for beta-globin to confirm presence of amplifiable DNA.

*Urine samples*: Participants were instructed by study staff on how to conduct a clean catch urine collection (Linda and Vorvick 2024) and the participant self-collected in the nearest restroom. Urine samples were transported to the laboratory, at 4 °C (on ice packs), where they were aliquoted and stored at − 80 °C.

### NNAL biomarker measurements

4-(methylnitrosamino)-1-(3-pyridyl)-1-butanone, or NNK, is a tobacco-specific N-nitrosamine that is found in tobacco and tobacco products. Exposure to NNK occurs via direct inhalation of tobacco smoke (from smoking of from second-hand exposure) as well as through oral absorption by smokeless tobacco products, or absorbed through exposure to the surface of the skin (Wei et al. 2016). NNK is a well-established carcinogen that is metabolized to NNAL, a stable, biomarker that can be measured in urine using well established standardized laboratory protocols. The biomarker assay is sensitive enough to detect tobacco exposure in smokers and in second-hand smokers and is tobacco-specific in that it is not a dietary component and is only found in the environment when there is tobacco smoke present.

Metabolism of NNAL was performed on each participant sample by the Nicotine and Tobacco Product Assessment Shared Resource (NicoTar) Laboratory at the Roswell Park Comprehensive Cancer Center, Buffalo, NY. The assessment involved determination of the concentrations of free and total NNAL concentrations using the modified method described by (Jacob et al. 2008). Briefly, 1-ml samples (urine, QCs, calibration standards, and control blanks) were transferred into test tubes. NNAL-d3 (internal standard, Toronto Research Chemicals, Toronto, Ontario, Canada) and sodium potassium phosphate buffer were spiked in the individual samples.

The samples for measurement of total NNAL (i.e. Free (NNAL) and Conjugated (NNAL-Gluc) analysis were incubated with β-glucuronidase (Sigma Lot # SLCC9103) enzyme at 37 °C for 12 h, whereas the samples for measuring free NNAL skipped the enzyme cleavage step. A series of liquid-liquid extraction and derivatization was applied to all the samples. The products were evaporated to dryness in a SpeedVac, reconstituted in methanol containing hydrochloric acid and then transferred to Total Recovery LCMS Vials (Waters Corp.) for analysis. NNAL analysis was performed using Waters Xevo^®^ TQ-XS Tandem Mass Spectrometer with ACQUITY UPLC I-Class Chromatography System (UPLC-MS/MS) using a modified method (Jacob et al. 2008). The concentration of NNAL-Gluc was calculated by subtracting concentration of free NNAL from the concentration of total NNAL (i.e. NNAL-Gluc = Total NNAL- Free NNAL). In addition, urinary creatinine was measured in all urine samples for NNAL normalization and urine flow correction. For normalization of Total NNAL and Free NNAL (pg/mg):

$$ {\mathrm{Normalized}}\;{\mathrm{Total}}\;{\mathrm{NNAL}} = {\mathrm{Total}}\;{\mathrm{NNAL}}/{\mathrm{creatinine}} \times 100 $$

$$ {\mathrm{Normalized}}\;{\mathrm{Free}}\;{\mathrm{NNAL}} = {\mathrm{Free}}\;{\mathrm{NNAL}}/{\mathrm{creatinine}} \times 100 $$

$$\begin{aligned}{\mathrm{Normalized}}\;{\mathrm{NNAL}} - {\mathrm{Gluc}} = {\mathrm{Normalized}}\;{\mathrm{Total}}\;{\mathrm{NNAL}} -{\mathrm{Normalized}}\;{\mathrm{Free}}\;{\mathrm{NNAL}}.\end{aligned} $$

Lastly, using these data, NNAL detox ratio was calculated (*NNAL detox ratio = Normalized NNAL-Gluc/Normalized Free NNAL*) for each study participant and was classified as: poor (ratio < 2), intermediate (ratios 2–5), and extensive (ratio > 5) tobacco metabolizers. In this analysis metabolizer phenotype was re-categorized as poor (ratio < 2) or extensive/intermediate (ratio ≥ 2) groups. Metabolizer classification cutoffs were based on (Bhat et al. 2011), who used the ratio of NNAL-glucuronides to free NNAL to define poor, intermediate, and extensive metabolizers in the context of a validated LC/MRM-MS assay. We applied these published thresholds to align with an established analytical framework and enhance comparability with existing literature. Glucuronidation is the primary detoxification pathway for NNAL excretion. A high NNAL ratio (Normalized NNAL-Gluc/Normalized Free NNAL) indicates more efficient metabolism, with a greater proportion of NNAL converted to its glucuronidated form (consistent with intermediate or extensive metabolizer phenotypes). In contrast, a lower ratio reflects reduced metabolic efficiency, with higher levels of free (unconjugated) NNAL and greater potential for prolonged carcinogenic exposure.

Samples were submitted to the (NicoTar) Laboratory in three batches, with batch 2 and 3 including 10% of the samples from the previous batch repeated as batch controls (i.e. batch #2 included 16 samples from Batch #1, and Batch #3 included 14 samples from Batch #2). Each batch also included 2 samples from participants who reported as non-smokers as negative controls. Each batch also included laboratory control blanks and calibration standards based on standard protocols. For samples that resulted in NNAL readings below the limit of detection, the limit of detection for that batch was divided by the square root of two (1.414) and was inputted as a value for these samples to calculate the NNAL detox ratio. This is a standard approach for previous studies (Bhat et al. 2011).

### Genotype data

*Genotyping*: was performed on all germline DNA samples using a customized genotyping array manufactured by Illumina which includes all probes from the InfiniumTM Global Screening Array v3.0 bead arrays (654,027 markers) and 11,377 markers we have identified as Ancestry Informative Markers (AIMs) (Boudeau et al. 2023).This genotyping array originated from a previously developed and validated comprehensive panel of African ancestry-informative markers to improve detection of population structure and enable more accurate investigation of ancestry-related disease risk (Boudeau et al. 2023). Our AIMs included Single Nucleotide Polymorphisms (SNPs) identified within 1 Mb of genes involved in addiction, DNA repair, metabolic activation and detoxification of carcinogens (Phase I and II pathway genes e.g. Cytochrome P-450(CYP) and Glutathione *S*-transferases), and efflux of drug, hydrophobic agents other agents found in cigarette smoke (Phase III pathway genes e.g. classes of ATP Binding Cassette (ABC) transporters). Our final SNP lists include 9,514 AIMs which were considered successful post analysis determined by Illumina standard and also have been successfully genotyped in all our samples. This analysis focused on Phase I, II and III AIMs only.

While race was collected and reported as self-identified, analyses were not conducted as direct comparisons between self-identified racial groups. The study was designed to evaluate genetic ancestry which was determined and calculated as Ancestry proportion for each patient sample. 223,637 SNPs shared between 1000G normal samples, and our cohorts were used for ancestry proportion analysis. We used YRI and CEU population from the 1000G project as reference(Genomes Project et al. 2015) for this estimation. Estimated individual ancestry proportions for all samples were calculated using ADMIXTURE (Alexander et al. 2009).

While sex was collected as self-identified, genotyped sex was determined and confirmed for each study sample via the proportion of calls on Y-loci. When there were sex discrepancies between self-reported and genotype sex, the genotype sex was reported.

### Functional analysis of SNPs

*Regulome Database*: To determine functional context to SNPs we generated Regulome dB Scores for each. The Regulome database annotates SNPs identified in functionally active regions of the genome based on functional genomic assays such as Transcription Factor (TF) ChIP-seq and DNase-seq (from the ENCODE database) as well as those overlapping the footprints and QTL data. Regulome scores range from 1 to 7 along with supporting available data. The lowest score of 1 indicates the higher the likelihood that the SNPs are functionally significant. Regulome scores are interpreted as follows: 1a = eQTL/caQTL + TF binding + matched TF motif + matched Footprint + chromatin accessibility peak; 1b eQTL/caQTL + TF binding + any motif + Footprint + chromatin accessibility peak; 1c eQTL/caQTL + TF binding + matched TF motif + chromatin accessibility peak; 1d eQTL/caQTL + TF binding + any motif + chromatin accessibility peak; 1e eQTL/caQTL + TF binding + matched TF motif; 1f eQTL/caQTL + TF binding / chromatin accessibility peak; 2a TF binding + matched TF motif + matched Footprint + chromatin accessibility peak; 2b TF binding + any motif + Footprint + chromatin accessibility peak; 2c TF binding + matched TF motif + chromatin accessibility peak; 3a TF binding + any motif + chromatin accessibility peak; 3b TF binding + matched TF motif; 4 TF binding + chromatin accessibility peak; 5 TF binding or chromatin accessibility peak; 6 Motif hit; 7 Other (Boyle et al. 2012; Dong et al. 2023).

*Molecular Modeling*: The only two SNPs that encode a missense change in amino acids were subjected to molecular modeling, a computational method that goes beyond the conventional analyses found in public databases to predict whether the given SNP will yield deleterious effects on protein function. This method not only accounts for the structural changes from sidechain substitutions, but also surface accessibility changes and the ensuing effects on protein assemblies. The SNP amino acid change was mapped onto the three-dimensional coordinates for the protein structure of the gene. Paying attention to the 3D location and molecular details helps to characterize the SNP based on the predicted ability to modulate the corresponding protein function of the gene. Using AlphaFold structure predictions we can make better pathogenicity predictions and rationalize its effect on protein function. Specific modeling methods are as follows:

*Structural Modeling of CYP3A5 Isoforms -* The canonical full-length human Cytochrome P450 3A5 protein (UniProt ID: P20815) structure was experimentally determined by X-ray crystallography (PDB ID: 5VEU), which includes the Heme cofactor essential for enzymatic activity. To model the CYP3A5*3 isoform resulting from the rs776746 polymorphism, which lacks the N-terminal 113 amino acids, AlphaFold3 (AF3) (Abramson et al. 2024) was employed to predict the structure of the truncated variant. Structural alignments and visualization were performed using ChimeraX (Meng et al. 2023).

*Modeling of ZNF789–TRIM28 Protein–Protein Interaction -* Protein sequences and domain boundaries for ZNF789 (UniProt ID: Q5FWF6) and KAP1/TRIM28 (UniProt ID: Q13263) were curated from UniProt. Initial AlphaFold3 modeling was conducted using full-length canonical sequences to identify potential interaction regions. Subsequent models focused on the KRAB domain of ZNF789 (residues 1–100) and the coiled-coil region of TRIM28 (residues 245–345), using AlphaFold3 and ColabFold (v1.5.5) (Mirdita et al. 2022) with increased sampling (*n* = 40 models per complex). Structural analyses, including super-positioning of protein complexes, analysis of interfaces and contacts, and figure preparation were done with ChimeraX (Meng et al. 2023).

*Variant Effect Prediction Using AlphaGenome* - To assess the potential regulatory impact of candidate SNPs, we performed in silico variant effect prediction using AlphaGenome(Avsec et al. 2026), a sequence-based deep learning model developed for predicting regulatory variant effects across multiple genomic modalities. For each candidate SNP, genomic coordinates, reference allele, and alternate allele were used as input to AlphaGenome. Variant effects were estimated by comparing model predictions from the reference and alternate allele sequences at the same genomic locus. The predicted effect score was calculated as the difference between alternate-allele and reference-allele predictions. AlphaGenome predictions were used as functional annotation evidence to support interpretation and evaluation of the regulatory activity of SNPs identified from Firth penalized logistic regression models.

### Statistical methods

*Univariate analyses*: Socio-demographic characteristics (such as race, sex, age, marital status, income, and education) of all recruited healthy smokers were summarized using descriptive statistics. Fisher’s exact tests were used to examine the associations between AIMs and poor metabolizer phenotype.

NNAL measurements such as normalized total NNAL, normalized free NNAL, normalized conjugated NNAL and NNAL ratio were summarized using descriptive statistics (such as mean and standard deviation) for the entire cohort as well as for various subgroups identified by age and gender. Our initial analyses revealed a significant amount of missing data for pack years (> 60% missing), however data for cigarettes per day was more complete with only ~ 8% missing. The Kruskal–Wallis test was used to assess the relationship between each NNAL variable and the sub-groups. All univariate tests were two-sided and used a 5% Type I Error to determine statistical significance. Computations were performed using the R language (R Core Team 2021).

*Multivariable analyses*: The analysis primarily focused on the 178 AIMs corresponding to the phase I, II and III genes; of these, one SNP contained a value for only one genotype and was excluded from further analysis. Genotype was coded as 0, 1 or 2 and was treated as a quantitative variable. Ancestry information (in the form of YRI ancestry proportion taking value between 0 and 1) was matched to clinical and genotype data. A multivariable penalized logistic regression model was fit to each AIM to determine the strength of association between metabolizer phenotype, as defined earlier, and genotype after adjusting for confounders such as age, sex, number of cigarettes smoked per day and assay batch. Statistical significance of the association was first assessed using nominal *p*-values. *P*-values were then adjusted for multiple comparisons using the Benjamini-Hochberg false discovery rate (FDR) method. SNPs with *p* < 0.05 and FDR ≤ 0.2 were considered statistically significant. SNPs with *p* < 0.05 but FDR > 0.2 were considered nominally significant but did not meet the predefined multiple testing threshold. In addition, SNPs with *p* ≤ 0.1 that did not meet the FDR threshold were considered suggestive and included for exploratory analyses. These findings are interpreted as hypothesis-generating. For each SNP, odds ratios (OR) and corresponding 95% confidence intervals (CI) were computed to further quantify the degree of association between metabolizer phenotype and genotype. Furthermore, a multivariable LR model was fit to determine the strength of association between metabolizer phenotype category and ancestry proportion after adjusting for confounders such as age, sex, number of cigarettes smoked per day and assay batch. Since the genetic variants evaluated are AIMs, and the primary objective is to examine the relationship between genetic ancestry and NNAL metabolizer phenotype, population structure is intrinsic to the exposure of interest rather than a confounder that can be fully adjusted for without attenuating the signal under investigation. Furthermore, the study population was predominantly composed of individuals of African ancestry (Table 1), reflecting continuous variation in admixture rather than discrete population subgroups. Therefore, we did not control for population stratification.

**Table 1:** Sociodemographic Characteristics of all recruited smokers (*n* = 274)

	Male, *n* = 131 *n* (%)	Female, *n* = 143 *n* (%)	Total, *N* = 274 *N* (%)
Marital status, *n* (%)			
Married/Domestic partnership	22 (16.8)	25 (17.5)	47 (17.2)
Never married	65 (49.6)	77 (53.8)	142 (51.8)
Divorced/separated/windowed	22 (16.8)	27 (18.9)	49 (17.9)
Unknown	22 (16.8)	14 (9.8)	36 (13.1)
Age, years, n (%)			
23–39	7 (5.3)	11 (7.7)	18 (6.6)
40–56	58 (44.3)	53 (37.1)	111 (40.5)
57–72	59 (45.0)	72 (50.3)	131 (47.8)
73–88	5 (3.8)	6 (4.2)	11 (4.0)
Unknown	2 (1.5)	1 (0.7)	3 (1.1)
Race, n (%)			
Black/African American	128 (97.7)	141 (98.6)	269 (98.2)
White	3 (2.2)	2 (1.4)	5 (1.8)
African Ancestry Proportions			
Range	0.003–0.977	0.002–0.946	0.002–0.977
Median ± SD	0.817 ± 0.158	0.830 ± 0.159	0.825 ± 0.158
Education, n (%)			
Less than 11 years	13 (9.9)	23 (16.1)	36 (13.1)
High School Graduate / G.E.D.	56 (42.7)	52 (36.4)	108 (39.4)
Associate degree/some degrees including vocational/technical school	28 (21.4)	43 (30.1)	71 (25.9)
Bachelor’s/graduate degree	12 (9.2)	9 (6.3)	21 (7.7)
Unknown	22 (16.8)	16 (11.2)	38 (13.9)
Income, n (%)			
Less than $14,999	19 (14.5)	31 (21.7)	50 (18.2)
$15,000 to $24,999	10 (7.6)	13 (9.1)	23 (8.4)
$25,000 to $49,999	15 (11.5)	12 (8.4)	27 (9.9)
$50,000 to $100,000	5 (3.8)	2 (1.4)	7 (2.6)
Unknown	82 (62.6)	85 (59.4)	167 (60.9)
Cigarette per day			
Less than 1	8 (6.1)	7 (4.9)	15 (5.5)
1 to 5	40 (30.5)	43 (30.1)	83 (30.3)
6 to 10	46 (35.1)	55 (38.5)	101 (36.9)
11 to 20	24 (18.3)	22 (15.4)	46 (16.8)
Over 20	3 (2.3)	3 (2.1)	6 (2.2)
Unknown	10 (7.6)	13 (9.1)	23 (8.4)
Packs per day			
Less than 0.25	37 (28.2)	36 (25.2)	73 (26.6)
0.25 to 0.499	34 (26.0)	45 (31.5)	79 (28.8)
0.5 to 0.99	29 (22.1)	37 (25.9)	6 (2.2)
More than 1	21 (16.03)	12 (8.39)	33 (12.0)
Unknown	10 (7.6)	13 (9.1)	23 (8.4)

## Results

### NNAL (tobacco) metabolism phenotypes among smokers

**Fig. 1:**
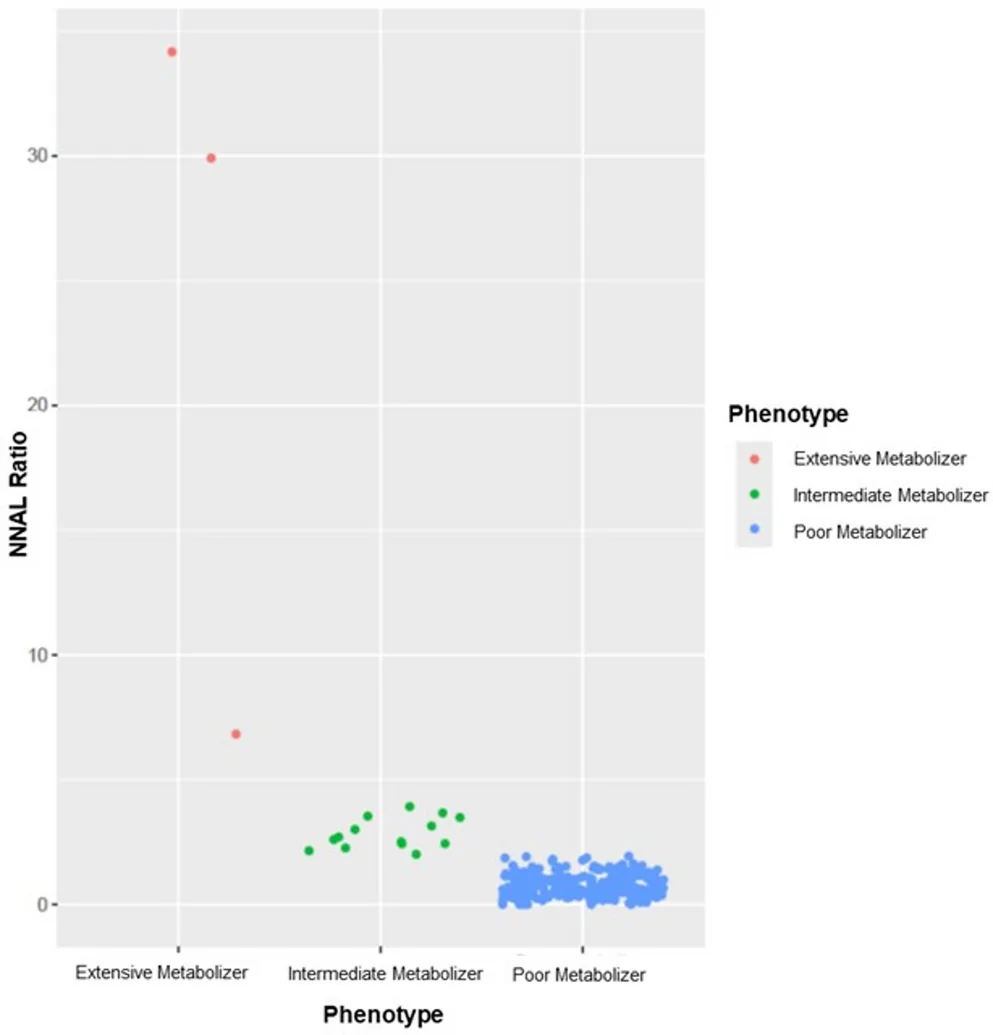
Distribution of NNAL metabolism by phenotype reveals that most of the study population were poor metabolizers of tobacco

Table 1 describes the characteristics of the study population. Urine samples were analyzed to evaluate NNAL metabolism efficiency. Three distinct metabolizer phenotypes were identified: poor metabolizers (*n* = 257, 94%) with a NNAL detox ratios < 2, intermediate metabolizers (*n* = 14, 5%) with a NNAL detox ratio between 2 and 5, and extensive metabolizers (*n* = 3, 1%) with a NNAL detox ratio > 5 (Fig. 1). Only 6% (16/269) of participants who self-identify as Black were found to be either intermediate or extensive metabolizers of NNAL. In contrast 20% (1/5) of participants who self-identify as White were found to be intermediate metabolizers of NNAL, however this difference was not statistically significant (*P* = 0.276). Similarly, a higher proportion of males, 8.4% (11/131) were found to be intermediate or extensive metabolizers of NNAL in comparison to 4.2% (6/143) of females who were found to be intermediate metabolizers of NNAL, but also not statistically significant (*P* = 0.210).

The median and standard deviation of NNAL detox ratios, stratified by age, sex (at birth), and packs per day, are presented in Table 2. There was no statistically significant difference in NNAL metabolic capacity according to age. As expected, as the number of packs per day smoked increases there is also a statistically significant increase in total, free and conjugated NNAL (*P* < 0.001, < 0.003 and < 0.001, respectively). However, NNAL metabolic capacity was not significantly different according to packs per day smoked. Despite males and females smoking similar number of cigarettes per day, female participants had a statistically significantly higher total, free and conjugated NNAL but their NNAL metabolic capacity was not significantly different from males (*p* = 0.469).

**Table 2:** NNAL metabolism by smokers age, gender, and pack years (*n* = 274)

	Normalized Total NNAL (pg/mg creatinine)	Normalized Free NNAL (pg/mg creatinine)	Normalized NNAL-Gluc (pg/mg)	NNAL-Gluc: NNAL Ratio
Median (s.d.)	90.75 (157.01)	49.3 (118.02)	37.9 (50.06)	0.71 (2.76)
Age				
< 52	79.65 (136.38)	48.65 (77.60)	32.15 (64.64)	0.67 (0.64)
52–57	92.60 (75.25)	52.50 (43.82)	34.60 (38.76)	0.74 (0.67)
58–62	90.10 (254.20)	48.90 (210.64)	38.50 (49.90)	0.60 (5.57)
63+	85.00 (126.73)	45.40 (87.14)	45.60 (45.33)	0.84 (0.95)
*p*-value	0.899	0.982	0.351	0.238
Sex				
Male	68.90 (68.22)	38.30 (45.94)	27.00 (29.53)	0.76 (3.93)
Female	116.10 (201.71)	65.50 (154.42)	47.10 (60.87)	0.68 (0.58)
*p*-value	< 0.001	< 0.001	< 0.001	0.469
Packs per day				
< 0.25	67.90 (73.78)	41.00 (49.22)	25.40 (31.54)	0.67 (4.02)
0.25–0.499	94.30 (83.54)	56.60 (53.43)	44.00 (37.04)	0.76 (3.32)
0.5–0.99	113.60 (268.96)	53.55 (213.67)	47.50 (70.19)	0.78 (0.50)
1 or more	124.80 (131.68)	76.50 (85.96)	45.70 (56.35)	0.85 (0.46)
*p*-value	< 0.001	0.003	< 0.001	0.637

### Genetic ancestry association with NNAL (tobacco) metabolism phenotypes

Among the AIMs (*N* = 177) corresponding to phase I, II, III pathway genes, univariate analyses identified a total of 14 SNPs with statistically significant associations with metabolizer phenotype (Fisher’s exact test *p*-value ≤ 0.05, see Supplementary Table 1).

A multivariable model was fit to examine the association of ancestry proportion with NNAL phenotype after adjusting for age, sex, number of cigarettes smoked per day and assay batch. Each 10% increase in African ancestry proportion was associated with a 29% increase in the odds of the poor metabolizer phenotype (OR = 1.29, 95% CI 0.94–1.69). However, the association was marginally significant (*p* = 0.075).

**Fig. 2:**
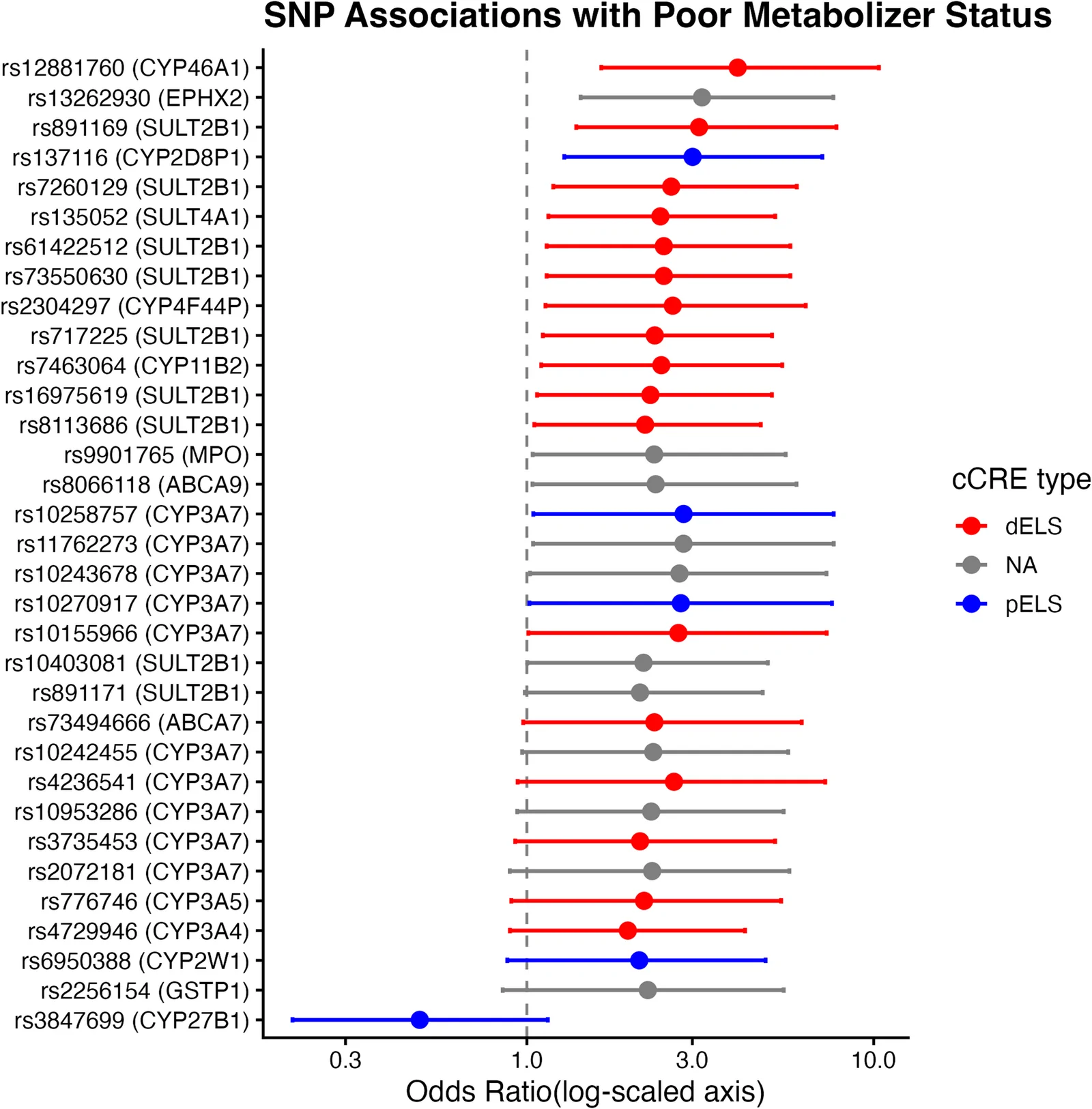
Forest plot of SNP associations with poor tobacco metabolizer status: Odds ratios (OR) and 95% confidence intervals are shown for 33 SNPs with nominal *p* ≤ 0.1 from logistic regression modeling poor metabolizer status (NNAL-glucuronide/NNAL ratio < 2) as the binary outcome, adjusting for age, sex, average cigarettes per day, and assay batch. The x-axis is log-scaled; the dashed vertical line at OR = 1 indicates no association. Point colors indicate overlap with candidate cis-regulatory elements (cCREs) from the ENCODE Registry: red, distal enhancer-like sequence (dELS); blue, proximal enhancer-like sequence (pELS); gray, no cCRE overlap (NA). SNP labels indicate rsID and the nearest gene in parentheses

To examine the association of phase I, II and III SNPs with metabolic capacity, after adjusting for age, sex, number of cigarettes smoked per day and assay batch, a total of 21 SNPs showed nominal significant association with metabolizer phenotype (*p*-value < 0.05) but did not survive multiple testing correction. (Fig. 2). The results for all 177 SNPs included in this analysis, along with the corresponding ORs and 95% CIs, are summarized in Supplementary Table 2.The majority of nominally significant SNPs (52%) were identified in Phase I (*N* = 11 SNPs in or within ±1 Mb of *CYP2D8P1*,* CYP3A7*,* CYP4F44P*,* CYP11B2*,* CYP46A1*,* EPHX2*, and *MPO*), Phase II (*N* = 9 SNPs in or within ±1 Mb of *SULT2B1* and *SULT4A1*) and Phase III (*N* = 1 SNP in or within ±1 Mb of *ABCA9)* pathway genes (Table 3). All SNPs identified showed an OR > 1 which suggests that a gain of one African allele may result in an increased odds of being a poor metabolizer of tobacco. This set includes 4 SNPs (rs12881760, rs13262930, rs135052 and rs7463064) identified to have a statistically significant association with phenotype in univariate analyses (based on Fisher’s exact test, see Supplementary Table 1) that did not account for any confounders. At a slightly loose threshold of *p*-value ≤ 0.1, there were 33 SNPs that showed an association with metabolizer phenotype in the multivariable analyses. This slightly larger set provides further room for exploration of the association between metabolizer phenotype and genotype (Table 3). Nominally significant associations were observed, and the direction and magnitude of these effects are consistent with the hypothesized relationship between metabolizer phenotype and genotype; however, these findings did not survive multiple testing correction and should be interpreted as exploratory. Functional analysis and bioinformatics mining of the identified SNPs is presented in the next section.

**Table 3:** Ancestry informative markers near tobacco metabolism genes are associated with tobacco metabolizer phenotypes. Top 21 SNPs with *p*-values ≤ 0.05 and the remaining *p*-values ≤ 0.1

rs ID	RegulomeDB rank score	Gene (rs ID Locus)	Nearest Phase I, II III Gene	Phase pathway
rs12881760	1f	DLK1	CYP46A1	Phase I
rs13262930	7	EPHX2	EPHX2	Phase I
rs891169	1d	ARHGEF1	SULT2B1	Phase II
rs137116	1f	RNU12	CYP2D8P1	Phase I
rs7260129	1b	LOC100505585	SULT2B1	Phase II
rs135052	2b	MPPED1	SULT4A1	Phase II
rs61422512	2b	LOC100505585	SULT2B1	Phase II
rs73550630	1f	ARHGEF1	SULT2B1	Phase II
rs2304297	2b	CHRNA6	CYP4F44P	Phase I
rs717225	1f	ARHGEF1	SULT2B1	Phase II
rs7463064	1f	FAM83H	CYP11B2	Phase I
rs16975619	1b	ARHGEF1	SULT2B1	Phase II
rs8113686	1f	ARHGEF1	SULT2B1	Phase II
rs9901765	1f	EPX	MPO	Phase I
rs8066118	1f	ABCA9	ABCA9	Phase III
rs10258757	1f	ARPC1B	CYP3A7	Phase I
rs11762273	1f	ARPC1B	CYP3A7	Phase I
rs10243678	1a	ARPC1B	CYP3A7	Phase I
rs10270917	1f	ARPC1B	CYP3A7	Phase I
rs10155966	1f	ARPC1B	CYP3A7	Phase I
rs10403081	1b	ARHGEF1	SULT2B1	Phase II
rs891171	1f	ARHGEF1	SULT2B1	Phase II
rs73494666	1b	MIDN	ABCA7	Phase III
rs10242455	1f	CYP3A5	CYP3A7	Phase I
rs4236541	1f	KPNA7	CYP3A7	Phase I
rs10953286	1b	MYH16	CYP3A7	Phase I
rs3735453	1f	ZSCAN25	CYP3A7	Phase I
rs2072181	1f	PDAP1	CYP3A7	Phase I
rs776746	1f	CYP3A5	CYP3A5	Phase I
rs4729946	1f	RELN	CYP3A4	Phase I
rs6950388	1f	UNCX	CYP2W1	Phase I
rs2256154	1f	LOC100130987	GSTP1	Phase II
rs3847699	1a	MBD6	CYP27B1	Phase I

### Functional analysis of significant AIMs

Among the nominally significant SNPs associated with NNAL metabolism phenotype (*P* < 0.05), the majority (95.2%, 20/21) had Regulome scores of 1 or 2 and among these, more than half (60%, 12/20) of the SNPs had a score of 1f suggesting functional activities such as eQTL/caQTL, TF binding and chromatin accessibility peak and One SNP (rs13262930) with a Regulome score of 7 was considered lower likelihood of being functional but was also noted as an eQTL. This SNP was among the five SNPs that were significantly associated with NNAL metabolizer Phenotype in the univariate and nominally significant in multivariable analyses. All 13 SNPs (*P* > 0.05 but ≤ 0.1) had a ranking of 1 with the majority 10/13 also having a score of 1 f.

To characterize the regulatory consequences of nominally significant non-coding variants, we applied AlphaGenome, a deep learning model that predicts allele-differential effects across transcription factor binding, histone modifications, chromatin accessibility, gene expression, and 3D chromatin contacts from a 1-Mb sequence window at base-pair resolution. Among all variants prioritized for functional annotation, rs12881760 (chr14:100,709,998 G > C) yielded the most mechanistically coherent signal. The variant is located adjacent to DLK1 within the chr14q32 imprinted cluster - a locus implicated in lung, liver, and hematologic malignancies(Enterina et al. 2017; Luk et al. 2011; Manodoro et al. 2014). AlphaGenome predicted allele-differential CTCF binding across approximately 20 cell types, accompanied by a bivalent chromatin signature (H3K4me1 + H3K27me3) consistent with an imprinting control region, and downstream perturbation of the locus’s non-coding RNA cluster (SNORD114-22, MIR770, SNORD113-4). To provide three-dimensional support, we examined AlphaGenome’s Hi-C contact differential predictions in IMR-90 normal lung fibroblasts - a smoking-relevant tissue context. The alternative allele showed a clear redistribution of local chromatin contacts centered on the variant position (Supplementary Fig. 1), consistent with disruption of a CTCF-dependent insulator boundary. The convergence of CTCF binding loss, bivalent chromatin remodeling, ncRNA dysregulation, and altered 3D contacts provides a mechanistically coherent basis for prioritizing rs12881760 as a candidate for functional follow-up.

**Fig. 3:**
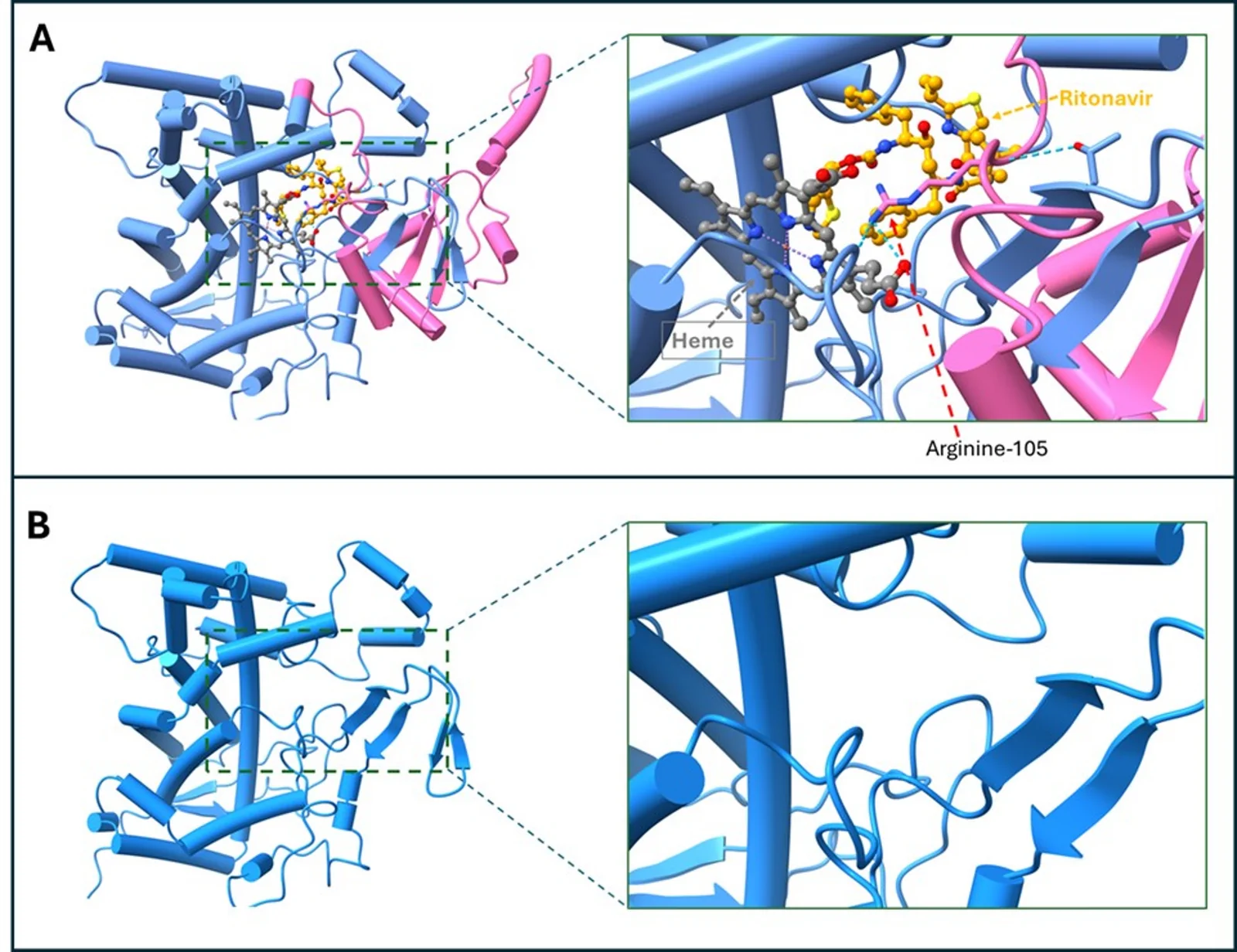
**(A)** CYP3A5-Isoform 1 from the known structure PDB code 5VEU. The known structure of CYP3A5 is shown in a cartoon representation where helices are shown as tube cylinders and beta-strands shown as flat arrows. The necessary Heme co-factor is shown in a gray ball-and-stick representation while the inhibitor *Ritonavir as orange ball-and-sticks*,* which marks the position where a typical substrate would bind in the active site (See discussion text for relevance of this structure shown here)*. The portion of the CYP3A5 protein shown in pink displays the first 113 residues found in isoform 1* but removed in isoform 3*. The right half of the figure zooms in on the active site region showing more structure details. Hydrogen bonds are shown with blue dashed lines. Arginine 105 (shown in pink and blue sticks) is making a hydrogen bond to the necessary Heme cofactor. Notably this residue along with others necessary for formation of the active site pocket are removed in isoform 3*. **(B)** CYP3A5-Isoform 3* AlphaFold 3 model. The predicted structure of CYP3A5 isoform 3* is shown in a cartoon representation as described above. The necessary Heme co-factor is not shown, as it is likely that removal of the first 113 residues (also missing the important Arginine-105) likely results in a protein that is defective in binding the heme cofactor. It is also unknown if this isoform is compromised for binding typical substrates. The right half of the figure zooms in on the active site region which is predicted to form a very similar structure to that found in the isoform 1 structure, albeit with an incomplete substrate pocket due to the missing residues. The loss of these residues in isoform 3* likely disrupts heme binding and active site formation, providing a structural basis for reduced enzymatic activity and altered NNAL metabolism

While all SNPs were located within 1 Mb of a Phase I, I, III gene, rs776746 and rs6962772 were the only significant SNPs (*p*-values ≤ 0.1), located within the protein-coding sequence of a Phase I gene (*CYP3A5)* and the transcription factor *ZNF789* gene (which is within 1 Mb of *CYP3A7)*, respectively. The structure of the Cytochrome P450 3A5 protein was previously determined by X-ray diffraction (PDB code 5VEU) and contains the necessary Heme co-factor bound in the active site that is required for catalytic activity(Hsu et al. 2018) (Fig. 3A). The *CYP3A5* polymorphism (rs776746, GG homozygous European ancestry variant) causes an alternative splicing isoform (CYP3A5*3) which results in a protein that removes the first 113 amino acids from the canonical full-length CYP3A5 protein (Fig. 3B). We used AlphaFold3 (AF3) (Abramson et al. 2024) to predict the structure of this N-terminal truncated CYP3A5*3 variant. While this variant has this large N-terminal truncation, the predicted model of the remaining residues reveals a protein that folds with very high similarity to the known structure of full-length CYP3A5, albeit missing important structural elements that contribute to the active site. This N-terminal truncation removes several key residues (i.e. R105) that are required for binding of the Heme cofactor. We predict this polymorphism would be severely deficient in catalytic activity, due to weaker binding interactions with the Heme co-factor.

**Fig. 4:**
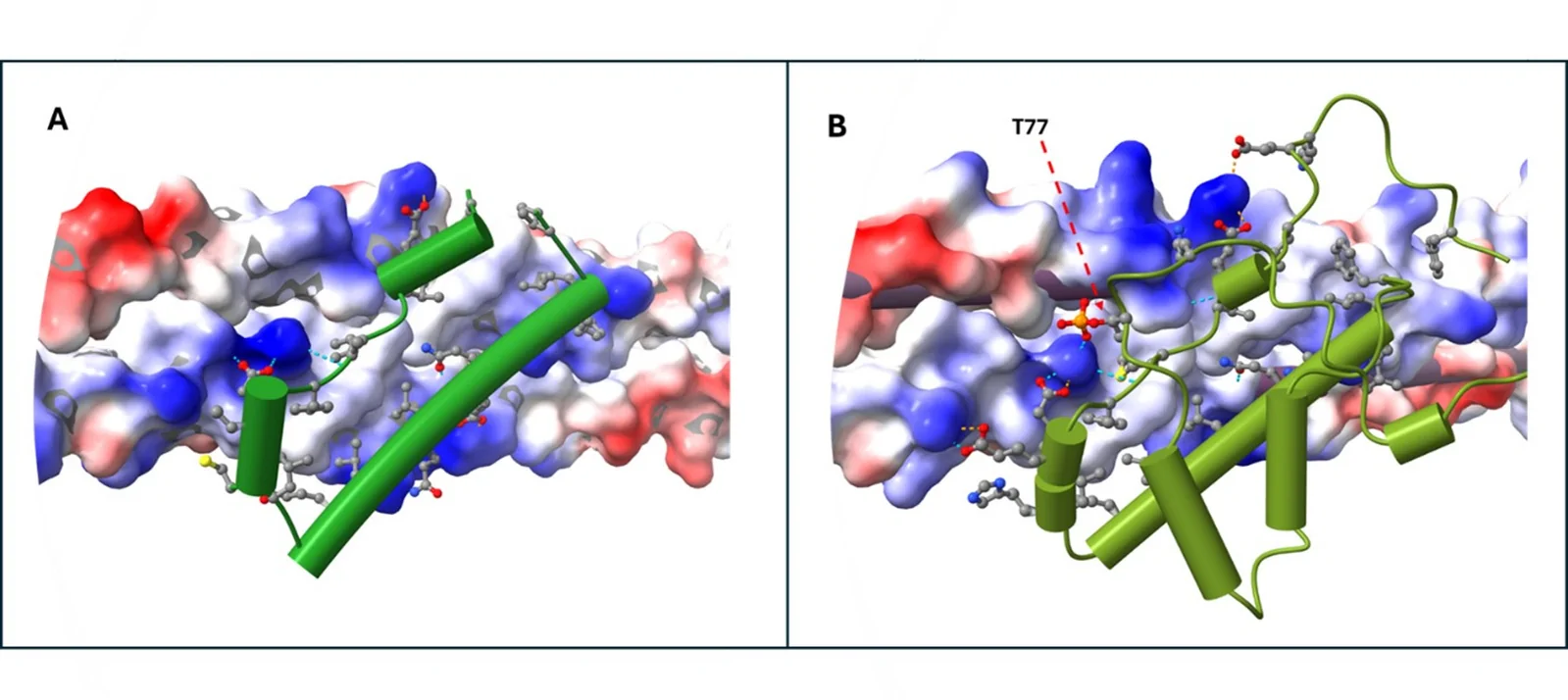
**(A)** ZNF93 complex with dimer of KAP1. The portion of the KAP1 dimer known to bind ZNF-KRAB domains (PDB code 7Z36) is shown in an electrostatic surface representation with blue (positively charged) and red (negatively charged) coloring. The ZNF93-KRAB domain (residues 1–71) is shown in green, and the residues involved in the interface are shown in ball-and-stick representation. Hydrogen bonds between the ZNF93 protein and KAP1 are shown as dashed blue or orange lines, and the interface formed has 1148 Å^2^ of buried surface area. (see details in the text). **(B)** Alphafold 3 model of ZNF789-T77 complex of ZNF789 with dimer of KAP1. The portion of the KAP1 dimer known to bind ZNF-KRAB domains is shown in an electrostatic surface representation with blue (positively charged) and red (negatively charged) coloring. The model of the ZNF789-KRAB domain (residues 1-100) is shown in olive green, and the residues involved in the interface are shown in ball-and-stick representation. The red dotted arrow shows residue T77 which is changed to an alanine for rs6962772. Its location is very near the interface with KAP1. T77 is portrayed as a phosphorylated residue which would then be able to contribute another hydrogen bond to the KAP1 protein. Hydrogen bonds are shown as dashed blue or orange lines, and the interface formed in this model is highly similar to the known structure in panel A (see details in the text). The proximity of the T77 residue to the KAP1 interaction interface suggests that this variant may influence protein-protein interactions involved in CYP gene regulation, providing a potential mechanistic link to altered NNAL metabolism

**Fig. 5:**
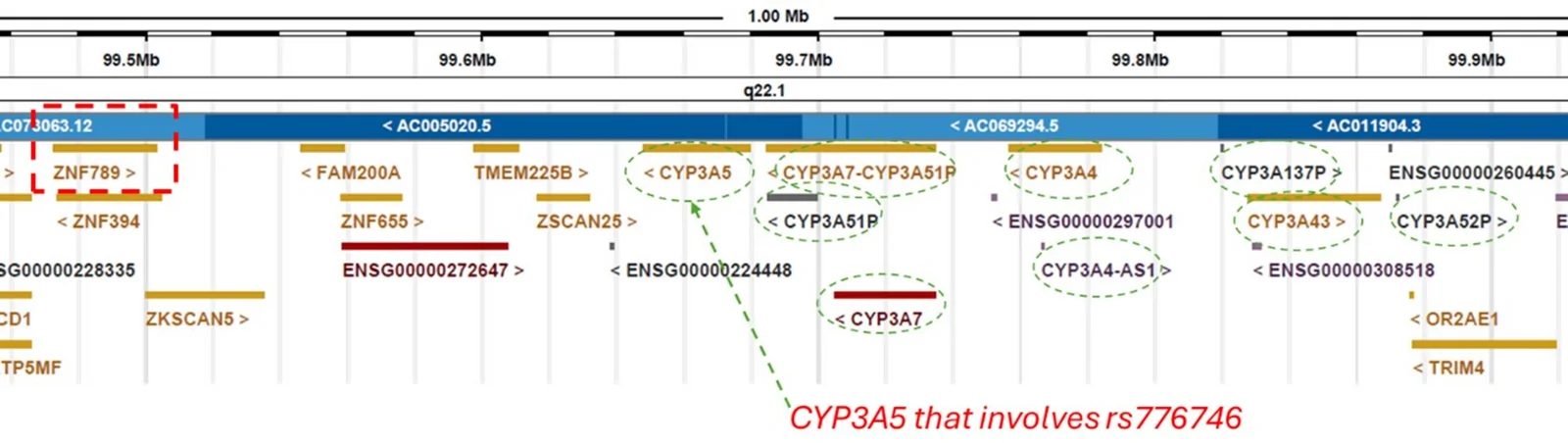
Genome browser view of the CYP Gene cluster relevant to the two SNPs highlighted in these studies (Chromosome 7:99,476,457 - ~99,900,000). CYP genes are circled with green ovals, and the green arrow highlights CYP3A5 that involves rs776746. The red dotted box highlights ZNF789 (contains rs6962772) just 0.2 Mb away. Coordinating gene expression within this cluster of metabolism genes may be an important theme in future studies

We also examined a non-significant associated SNP (Odds Ratio: 1.97, 95% CI 0.75–4.83), rs6962772 that results in missense T77A aa change in unstructured loop of the N-terminal KRAB domain of *ZNF789* gene. Most missense predictors (including AlphaMissense) predict this allele to be benign. However, changing a Threonine to Alanine could remove the potential for phosphorylation and phospho-regulation. The human Zinc finger protein 789 (UniProt: Q5FWF6) is known to bind to KRAB-associated protein 1 (KAP1) (also known as Tripartite motif-containing protein 28 (TRIM28), Transcription intermediary factor 1‑β (TIF1β) or KRAB‑interacting protein 1 (KRIP‑1) (Q13263). We sought to understand how these two proteins interact with each other, and how rs6962772 might affect that interaction. Protein sequences and domain boundaries for both proteins were refined from Uniprot entries. AlphaFold3 (AF3) modeling was first performed with full-length canonical protein sequences from Uniprot, to infer the regions of each protein that were predicted to interact. This first phase identified the KRAB domain of ZNF789 (residues 1 to 100) binding to a long alpha helix in KAP1 (residues 245–345). To optimize AF3 modeling and scores, subsequent models were built with these specific protein sequences. The resulting models were predicted with strong confidence scores (ipTM range 0.67 to 0.70, and ipSAE range 0.52 to 0.56) (Dunbrack 2025). There is a known structure of another KRAB domain (PDB: 7Z36, ZNF93 Uniprot: P35789) bound to this exact same position on KAP1 (Fig. 4A). Superposition of the AF3 model on the known structure reveals a highly similar conformation (RMSD between 42 alpha carbon atom pairs is 0.59 Angstroms for the KRAB domains, and RMSD between 37 alpha carbon atom pairs is 0.53 Å for the KAP1 helix). It is very interesting that we observe the SNP (rs6962772) residue reported in this study (ZNF789:T77A) is located at the interface between the KRAB domain and the KAP1 helix (Fig. 4B). For more quantitative scoring of complexes, the ColabFold version (1.5.5) (Mirdita et al. 2022) of AlphaFold2 was used with higher sampling (40 models for each protein complex) and ipTM scoring ranges for specific chain pairs were compared between the wild-type residue ZNF789:T77, and the SNP residue ZNF789:T77 to A to see how the SNP might change the interaction. It is possible that rs6962772 could affect how well ZNF789 and KAP1 proteins interact. We also examined the “genomic neighborhood of *ZNF789* and noted that *ZNF789* maps within 0.2 Mb of our other gene (*CYP3A5*) which was also noted to carry a SNP nominally significantly associated with NNAL metabolism and at least 9 other CYP genes are within 0.35 Mb of *ZNF789* (Fig. 5).

Table 4 shows the mean NNAL ratios according to SNP genotypes for rs776746 and rs6962772. Persons with the CYP3A5 *3 genotype (GG) which is homozygous with no African Ancestry alleles had a mean NNAL ratio of 2.06 and are categorized as intermediate metabolizers of NNAL. In contrast persons heterozygous or homozygous with the African Ancestry Alleles (AG and AA) had lower NNAL ratios and categorized as poor metabolizers (1.16 and 0.86 respectively). For rs6962772, an increase in mean NNAL ratio according to genotype was not observed.

**Table 4:** Mean NNAL ratios according to genotype for rs776746 (CYP3A5) and rs6962772 (ZNF789)

Genotype	African ancestry alleles	*N*	NNAL-Gluc: NNAL ratio mean ± SD	Metabolizer phenotype
rs776746 (CYP3A5)				
GG (CYP3A5*3)	0	30	2.06 ± 6.11	Intermediate
AG (CYP3A5 *1/*3)	1	107	1.16 ± 2.91	Poor
AA (CYP3A5 *1)	2	137	0.86 ± 0.70	Poor
rs6962772 (ZNF789)				
AA	0	19	1.06 ± 0.83	Poor
AG	1	70	1.35 ± 4.07	Poor
GG	2	185	1.02 ± 2.24	Poor

## Discussion

Most tobacco carcinogens, including NNK and NNAL are metabolized by various enzymes or enzyme families, leading to metabolic activation catalyzed by phase I enzymes such as CYP-450s that convert these carcinogens to forms that can covalently bond to DNA to form adducts. Activated carcinogens further undergo metabolic detoxification catalyzed by phase II enzymes such as glutathione-*S*-transferases (GSTs), uridine-5′-disphosphate-glucuronosyltransferases (UGTs) and others resulting in excretion of conjugated carcinogen metabolites in generally harmless forms (HHS 2010). In addition, phase III genes include several classes of ATP Binding Cassette (ABC) transporters directly efflux NNK and NNAL metabolites found in cigarette smoke, from cells by active transport mechanisms (Leslie et al. 2001). ABC transporters facilitate excretion of phase II conjugated products by transporting these products outside the cell (Leslie et al. 2001). It is the balance between metabolic activation, detoxification and transport of tobacco carcinogens that contributes to the risk of smoking-related cancers among smokers.

This study reveals insights into tobacco metabolism patterns among smokers, particularly highlighting racial differences in NNAL metabolism. Our data suggests that most smokers in this cohort may carry a biologic liability to retain NNAL, a carcinogenic metabolite of the tobacco nitrosamine NNK. Most of this cohort had high proportion of African ancestry (median African ancestry 82%). Consistent with the observed association between African ancestry and poor NNAL metabolizer phenotype, it was therefore not surprising to observe that 94% of this cohort classified as poor metabolizers. We used metabolizer classification cutoffs based on (Bhat et al. 2011), who defined poor, intermediate, and extensive metabolizers using the ratio of NNAL glucuronides to free NNAL within a validated LC/MRM-MS assay. These thresholds were applied to align with an existing analytical framework and enhance comparability with prior literature. The high proportion of participants classified as poor metabolizers likely reflects both the application of these predefined cutoffs and the underlying characteristics of our study population, which is predominantly of African ancestry. Although these findings may suggest differences in NNAL metabolic profiles, direct comparisons with prior studies are limited by differences in population characteristics and analytical approaches, as many studies report continuous measures of glucuronidation rather than categorical metabolizer classifications. As such, variation in observed distribution of metabolizer phenotypes across studies may reflect differences in methodology, population characteristics and the application of classification thresholds. These smokers may experience higher and more prolonged internal NNAL exposure per cigarette. Because NNAL persists systemically and forms DNA adducts, a slower metabolic clearance plausibly raises effective carcinogen dose at target tissues (e.g., lung epithelium or oral mucosa) even when individuals report similar smoking behavior. In practical terms, equal cigarette consumption may not correspond to equivalent biological exposure: poor metabolizers may accrue a higher carcinogenic burden for the same behavior. In addition to the role of metabolic activation and detoxification, impaired NNAL metabolism may also contribute to downstream biological processes relevant to carcinogenesis, including oxidative stress and inflammation. NNAL and related tobacco carcinogens have been shown to induce oxidative damage and inflammatory responses, which can further promote DNA damage, cellular dysfunction, and tumor development (Caliri et al. 2021; Li and Hecht 2022). Reduced metabolic clearance of NNAL may prolong systemic exposure, thereby increasing the potential for these downstream effects. Although these pathways were not directly evaluated in this study, they represent plausible mechanisms linking variation in NNAL metabolism to cancer risk and warrant further investigation.

Although our race-stratified comparison did not reach statistical significance (limited by sample size), the pattern that fewer intermediate/extensive metabolizers were found among Black participants (6%) than White participants (20%), is consistent with prior biomarker studies reporting greater nitrosamine exposure per cigarette among some Black smoker populations (Muscat et al. 2005; Rostron 2013). Similarly, we observed higher proportion of poor metabolizers among females compared to males and if replicated, this has two implications. First, biologic susceptibility may contribute to population-level cancer disparities alongside well-documented social and commercial determinants (e.g., menthol marketing, stress, access to cessation and screening). Second, risk models that rely only on pack-years may systematically underestimate risk in groups enriched for poor NNAL metabolizers.

Our genetic ancestry analysis identified SNPs nominally significantly associated with metabolizer phenotype. All of these variants (OR > 1) suggest that African ancestry-associated alleles linked to increased odds of being a poor metabolizer. Many of these variants are located in or near genes involved in Phase I metabolic pathways, particularly the CYP-450 enzyme system, which plays a central role in NNAL bioactivation.

The literature demonstrates that *CYP3A5* polymorphism (rs776746) results in an alternative splicing isoform (CYP3A5*3) that produces a truncated protein lacking the first 113 amino acids (Kuehl et al. 2001). This structural alteration removes key residues required for heme cofactor binding and is likely to reduce catalytic activity. Because *CYP3A5* is involved in Phase I metabolism of tobacco-related xenobiotics, including NNAL, reduced catalytic activity may influence the balance between NNAL bioactivation and detoxification pathways. The full-length canonical *CYP3A5* (CYP3A5*1 and CYP3A5*1/*3) may play an important role in NNAL metabolism, with catalytic function associated with higher NNAL ratios. We observed that smokers with the CYP3A5*1 (AA-homozygous for the African allele) and CYP3A5*1/*3 (AG – heterozygous for the African allele) genotypes have lower NNAL-gluc: NNAL ratios compared to those with the CYP3A5*3 (GG-homozygous for the European allele with the truncated CYP3A5*3 which is catalytically compromised) genotype. The increased NNAL-gluc: NNAL ratios associated with rs776746 genotype (GG – homozygous for the European allele with the truncated CYP3A5*3; see Table 4) suggest that CYP3A5 variation may influence NNAL metabolic processing, potentially affecting the balance between bioactivation and detoxification pathways. Given the higher frequency of the AA and AG genotypes in the Black population compared to the White population (Kuehl et al. 2001), this molecular evidence provides a potential biological mechanism that may contribute to population differences in tobacco-related cancer risk. A study conducted by Jiang et al. already shows that *CYP3A5* rs776746 polymorphism and smoking may influence the prognosis of NSCLC patients (Jiang et al. 2016). Further investigation of the role of this genetic ancestry polymorphism is critical for understanding tobacco-associated cancer risk.

Molecular modeling also reveals potential functional significance for one of the marginally associated SNPs (rs6962772). This ancestry informative marker is noted to be in the N-terminal KRAB domain of *ZNF789* gene. Kruppel-associated box (KRAB) zinc-finger proteins (KRAB-ZFPs) are transcriptional/transcription-regulatory factor family (Sun et al. 2022). It has been demonstrated that ZNF789 proteins interact with KAP1 to regulate CYP gene clusters (Bojkowska et al. 2012). Because CYP enzymes play a central role in Phase I metabolism of NNAL, variation in regulatory pathways influencing CYP gene expression may have downstream effects on NNAL metabolism. Therefore, as the ZNF789-T77A SNP (rs6962772) is found near the predicted interface with KAP1 in our AF3/AF2 models, this provides a connection of the rs6962772 to dysregulation of CYP proteins that are required for metabolism of tobacco related xenobiotics like NNAL. We hypothesize that wildtype ZNF789 cooperates with KAP1 to repress CYP gene expression, thus resulting in low efficiency in activating the NNAL molecule. If the interface between ZNF789 and KAP1 is compromised, there could be less repressive binding of both proteins to CYP gene promoters. In this way the T77A SNP (rs6962772) could influence CYP gene expression, potentially leading to altered NNAL metabolism and differences in NNAL-DNA Adduct formation. We did not observe a correlation between rs6962772 genotype and NNAL ratios. These findings should therefore be interpreted as hypothesis-generating. It is currently unknown whether ZNF789 bind specific CYP gene promoters. However, studies show that knocking out KAP1 has been shown to activate some CYP promoters and repress others; also, sex-related differences of activation verses repression have been shown at the same gene promoter (Bojkowska et al. 2012; Randolph et al. 2022). Nevertheless, these preliminary findings warrant validation in larger studies and further investigation. Overall, our findings from computational modeling and AlphaGenome evaluation suggest three potential mechanisms by which these variants may act: an allelic effect on a protein catalytic activity (rs776746), repression of gene expression (rs6962772) and redistribution of local three-dimensional DNA contacts (rs12881760). This should be interpreted as hypothesis-generating and experimental validation in future studies will be required to confirm the proposed functional mechanisms.

Our findings suggest implications for cancer risk assessment and personalized prevention strategies that can be further investigated. The association between genetic ancestry and metabolizer status suggests that genetic screening might help identify individuals at higher risk for tobacco-related cancers. The particularly high prevalence of poor tobacco carcinogen metabolizers among Black smokers may partially explain the documented disparities in lung cancer incidence and mortality. A key strength of our study is its comprehensive approach, combining genetic analysis with metabolic phenotyping. The inclusion of both Phase I, II, and III pathway genes provides a more complete picture of tobacco metabolism variation. However, our study had some limitations in sample size, particularly for White participants, which affected the statistical power to detect significance of some racial comparisons. Because participants were recruited through community- and clinic-based strategies rather than probability sampling, the study cohort may not be fully representative of the target population, and selection bias cannot be ruled out. While complete pack-year data were not available, our primary focus was on NNAL metabolism among current smokers, which is more directly influenced by current smoking status than cumulative exposure. Nonetheless, we acknowledge that variability in smoking history (e.g. duration) may not have been fully captured. In addition, self-reporting the number of cigarettes per day may have also skewed true packs per day data. Because ancestry-informative markers were the primary variables of interest, population structure was treated as part of the biological signal under investigation rather than solely as a source of confounding. We acknowledge that residual population stratification may still influence associations therefore, findings are interpreted cautiously and framed as hypothesis-generating.

### Future directions

Future studies should first validate these genetic signals in large, multi-ancestry cohorts to refine effect sizes and generalizability. Mechanistic and translational work is also needed to test interventions that modulate the implicated metabolic pathways—ideally tailored to ancestry or metabolizer phenotype. For example - the identified molecular mechanisms, particularly involving *CYP3A5* and related pathways, offer potential targets for therapeutic intervention and risk modification strategies. In Fig. 3A we show the molecule Ritonavir as orange ball-and-sticks, which marks the position where a typical substrate would bind in the active site. Ritonavir is an anti-retroviral drug that has been shown to be a potent inhibitor of CYP3A enzymes, including CYP3A5 (Loos et al. 2022) and therefore may serve as a potential therapy for reducing NNAL ratios among current smokers as part of a chemo preventive strategy used alongside tobacco cessation. Further investigations are needed to confirm this potential.

In parallel, pragmatic biomarker/genetic screening approaches should be developed and evaluated to identify smokers at elevated carcinogen burden for targeted prevention and surveillance. Finally, implementation research should determine how metabolizer-informed risk feedback can be embedded in smoking-cessation programs to improve quit rates and narrow disparities. These results substantiate the need for personalized approaches to cancer prevention and underscore the importance of considering genetic ancestry in assessing tobacco-related cancer risk.

## Supplementary Information

Below is the link to the electronic supplementary material.

Supplementary Material 1

Supplementary Material 2

Supplementary Material 3

Supplementary Material 4
